# Antibacterial activity and compositional analysis of *Acer truncatum* bunge leaf extract against *Staphylococcus aureus*

**DOI:** 10.17221/65/2024-VETMED

**Published:** 2025-05-28

**Authors:** Fei Liao, Jie He

**Affiliations:** Guizhou Vocational College of Agriculture, Qingzhen, P.R. China

**Keywords:** antimicrobial agents, cell membrane integrity, LC-MS analysis, phenolic compounds

## Abstract

*Staphylococcus aureus* is a zoonotic pathogen that can cause various diseases in both humans and animals. This experiment evaluates the antibacterial activity of extracts from *Acer truncatum* leaves against *S. aureus*, including the minimum inhibitory concentration (MIC), growth curve, and cell membrane integrity assessment, alongside the identification of the extract components by LC-MS. Results demonstrated that the MIC of the *n*-butanol extract from the ethanol extract of *Acer truncatum* leaves against *S. aureus* was 3.125 mg/ml, with a minimum bactericidal concentration (MBC) of 6.25 mg/ml. Over 16 h, the extracts at concentrations of 0.25 MIC, 0.5 MIC, and 1 MIC effectively inhibited *S. aureus* growth. The fluorescence staining revealed that the extracts at different concentrations compromised the structural integrity of the cell membrane. The LC-MS analysis identified the principal constituents of the extract as betaine (27.189%), 3,4-dihydroxymandelic acid (16.112%), quercitrin (14.768%), chlorogenic acid (8.778%) and neochlorogenic acid (4.452%). The study indicated that *Acer truncatum* leaf extract has good potential for application in natural antibacterial drugs.

As the global issue of antimicrobial misuse continues to escalate, increasing numbers of pathogenic bacteria are developing resistance, making the research and development of novel antimicrobials increasingly urgent (Gao et al. 2019). Natural products, serving as a rich source of antimicrobial agents, have gradually become a research focus due to their unique antibacterial mechanisms and reduced risk of drug resistance (Ekiert and Szopa 2020). *Acer truncatum* Bunge, also known as the Chinese maple, is a deciduous tree belonging to the *Aceraceae* family and the genus *Acer*. Classified under the genus *Acer* within the *Aceraceae* family, *Acer truncatum* is a native woody tree species in northern China, Japan, and the Korean Peninsula. Currently, it is widely cultivated globally for its ornamental and oil-producing values (Li et al. 2024). The leaves of *Acer truncatum* Bunge contain nutrients, such as protein, vitamins, and dietary fibre, alongside bioactive substances including flavonoids, chlorogenic acid (Fan et al. 2018), nervonic acid (He et al. 2020), and polysaccharides. These components have exhibited significant potential for antibacterial and anti-inflammatory applications (Lan 2019). Pharmacological studies indicate that *Acer truncatum* Bunge possesses diverse biological activities, such as acetylcholinesterase inhibition, antibacterial, antioxidant, antitumor, and fatty acid synthase inhibition (Fan et al. 2022).

*Staphylococcus aureus* (*S. aureus*), as a significant zoonotic bacterium, poses a serious threat to the health of humans and livestock on a global level (Deng et al. 2021; Troscianczyk et al. 2023). Practically any species of warm-blooded animals can act as healthy carriers of *S. aureus* or become similarly infected (Zhou et al. 2024). *S. aureus* is a key causative agent of contagious intramammary infections in dairy cattle (Zaatout et al. 2020). Its prevalence in milk samples was 34.4% (89/259) (Deepak et al. 2024). Additionally, the widespread misuse of antibiotics has accelerated the emergence of antibiotic-resistant strains of *S. aureus*, particularly methicillin-resistant *S. aureus* (MRSA), exacerbating the threat posed by this pathogen (Tasneem et al. 2022). MRSA infections are now classified among the three most challenging infectious diseases to treat worldwide. The thick peptidoglycan-teichoic acid cell wall and unique membrane architecture of* S. aureus* (gram-positive bacteria) function as dual effectors: the cell wall acts as a physical barrier against antibiotic penetration, whereas the membrane mediates adaptive responses, collectively enabling its notorious multidrug resistance (Tan et al. 2022). Consequently, controlling *S. aureus* necessitates heightened vigilance and evidence-based strategies, including the rational use of antibiotics, improved environmental hygiene, and enhanced personal hygiene practices, to mitigate its impact on humans and animals. Thus, identifying novel agents with potent antibacterial activity against *S. aureus* is critical for infections caused by this pathogen.

This study aims to investigate the antibacterial activity of *Acer truncatum* leaf extracts against *S. aureus*, validate their efficacy in antibacterial experiments, and preliminarily elucidate their mechanism of action. This research is anticipated to provide new candidate compounds for antibacterial drug development while advancing the utilisation of *Acer truncatum*, a traditional medicinal plant.

## MATERIAL AND METHODS

### Materials

The *S. aureus* [CMCC(B)26003] strain was purchased from Shanghai Luwei Technology Co., Ltd. (Shanghai, P.R. China). A casein peptone (MH) broth medium and a Luria broth (LB) agar medium were obtained from Qingdao Haibo Technology Co., Ltd. (Qingdao, P.R. China). Propidium iodide (PI) was purchased from Beijing Solarbio Science & Technology Co., Ltd. (Beijing, P.R. China). Anhydrous ethanol, ethyl acetate, ether, and *n*-butanol (analytical purity) were purchased from Shanghai Aladdin Biochemical Technology Co., Ltd. (Shanghai, P.R. China).

### Plant material and extraction

*Acer truncatum* Bunge materials were collected in June 2023 from Qinzheng County, Guizhou Province, P.R. China (26.55°N, 106.38°E, altitude: 1 256 m), and identified as *Acer truncatum* Bunge by Guangyao Tao of Guizhou Vocational College of Agriculture. Exactly 100 g of shade-dried *Acer truncatum* leaves (harvested in June) were crushed and then subjected to ultrasonic extraction with 60% ethanol for 1 h (liquid-to-solid ratio 1 : 15, 50 °C, 200 W power), and were then repeated 3 times. The mixture was filtered while hot and combined with the filtrate. The crude extract was obtained by rotary evaporation and concentration, then the crude extract was dispersed with 200 ml of water. It was sequentially extracted with equal volumes of petroleum ether, ethyl acetate, and saturated *n*-butanol; the upper organic phase was collected, rotary evaporated, and concentrated into a paste. It was then stored at 4 °C for future use.

### Antimicrobial susceptibility test of extracts

Using the paper disk diffusion method (K-B method) (Wang et al. 2020), 20 mg of extract from each extraction phase (petroleum ether, ethyl acetate, and saturated *n*-butanol) of *Acer truncatum* leaves were weighed and added to 200 μl of ethanol for ultrasonic dissolution. The dissolved extracts were then added to ampoules containing 10 pieces of antibiotic sensitivity disks and dried in a vacuum drying oven at 40 °C. Round disks (6 mm) containing 2 mg of extract were placed on the MH agar plates inoculated with a standard amount of *S. aureus* and incubated at 37 °C for 24 hours. The diameter of the inhibition zone was measured using vernier callipers.

### Minimum inhibitory concentration (MIC) assay

In this experiment, the *n*-butanol extraction phase with lighter colour, easy dissolution, and certain antibacterial activity was selected for further experiments. Using the two-fold dilution method, 100 μl of culture medium was added to wells 2 to 8 in a 96-well plate. 200 μl of the extract solution was added to well 1, and 100 μl of the sample solution was aspirated from well 1 to well 2, and so on, for serial dilutions up to well 8. 10 μl of the bacterial suspension with a concentration of 1 × 10^5 CFU/ml was added to wells 1 to 8. An equal amount of culture medium, bacterial suspension, and physiological saline was added to well 9 as the control group. Only culture medium was added to well 10 as the blank control. After sample loading, the plates were incubated at 37 °C for 24 hours. The MIC was determined based on a visual inspection of the solution turbidity and optical density OD_600 nm_ in each well. Three replicates were set for each group.

### Minimum bactericidal concentration (MBC) assay

Based on the MIC test results, 10 μl of the solution from each well was aspirated for plating and then incubated at 37 °C for 24 h to observe the growth of colonies on the agar plates. The lowest extract concentration without colony growth was considered the MBC of the *Acer truncatum* extract.

### Extract’s effect on the *S. aureus* growth curve

Bacterial suspensions of 100 μl (1~2 × 10^7 CFU/ml) were added to a 96-well cell culture plate. Subsequently, 100 μl of the extracts at concentrations of 2 MIC, 1 MIC, and 0.5 MIC were added to the wells, resulting in final concentrations of 0.25 MIC, 0.5 MIC, and 1 MIC. The culture medium was used in place of the extract as a control. Four wells were prepared for each concentration. The 96-well cell culture plate was incubated at 35 °C for 16 h, and the OD_600 nm_ was measured every 2 hours. Growth curves were plotted with time on the horizontal axis and optical density on the vertical axis.

### Extract’s effect on the *S. aureus* cell membrane integrity

Using the propidium iodide (PI) staining method, 500 μl of the bacterial suspension was taken into a 2 ml centrifuge tube, and then 500 μl of the extract solution with concentrations of 1 MIC, 2 MIC, and 4 MIC was added to the centrifuge tubes to achieve final concentrations of 0.5 MIC, 1 MIC, and 2 MIC, respectively. Sterile deionised water was used in place of the extract as a control. Three tubes were prepared for each concentration. The cultures were incubated at 28 °C for 4 hours. After incubation, the bacterial suspensions were centrifuged at 2 236 *g* at 4 °C for 10 minutes. The supernatant was discarded, and the bacterial pellets were resuspended and washed twice with phosphate-buffered saline (PBS). After centrifugation and discarding the supernatant again, the bacteria were resuspended in PBS and stained with PI (final mass concentration of 10 μg/ml). The stained bacteria were incubated in the dark at 4 °C for 30 minutes. Then, they were resuspended and washed twice with PBS. After centrifugation and discarding the supernatant, the bacteria were resuspended in PBS again. A 10 μl aliquot of the bacterial suspension was taken and observed under a fluorescence microscope. The excitation wavelength was 535 nm, and the emission wavelength was 615 nm (Giao et al. 2009; Liao et al. 2024).

### Identification of the chemical composition of the extracts

The extracts were analysed by Liquid Chroma-tography-Mass Spectrometry (LC-MS).

### Liquid chromatography conditions

The LC analysis was performed on a Vanquish UHPLC System (Thermo Fisher Scientific, Waltham, USA). Chromatography was carried out with an ACQUITY UPLC^®^ HSS T3 (2.1 × 100 mm, 1.8 μm) (Waters, Milford, MA, USA). The column was maintained at 4 °C. The flow rate and injection volume were set at 0.3 ml/min and 2 μl, respectively. For LC-ESI (+)-MS analysis, the mobile phases consisted of (B2) 0.1% formic acid in acetonitrile (v/v) and (A2) 0.1% formic acid in water (v/v). Separation was conducted under the following gradient: 0~1 min, 8% B2; 1~8 min, 8%~98% B2; 8~10 min, 98% B2; 10~10.1 min, 98%~8% B2; 10.1~12 min, 8% B2. For LC-ESI (–)-MS analysis, the analytes were carried out with (B3) acetonitrile and (A3) ammonium formate (5 mM). Separation was conducted under the following gradient: 0~1 min, 8% B3; 1~8 min, 8%~98% B3; 8~10 min, 98% B3; 10~10.1 min, 98%~8% B3; 10.1~12 min, 8% B3 (Zelena et al. 2009).

### Mass spectrum conditions

The mass spectrometric detection of the metabolites was performed on an Orbitrap Exploris 120 (Thermo Fisher Scientific, Waltham, USA) with an ESI ion source. Simultaneous MS1 and MS/MS (Full MS-ddMS2 mode, data-dependent MS/MS) acquisition was used. The parameters were as follows: sheath gas pressure, 40 arb; aux gas flow, 10 arb; spray voltage, 3.50 kV and –2.50 kV for ESI (+) and ESI (–), respectively; capillary temperature, 325 °C; MS1 range, m/z 100–1 000; MS1 resolving power, 60 000 FWHM; number of data dependant scans per cycle, 4; MS/MS resolving power, 15 000 FWHM; normalised collision energy, 30%; dynamic exclusion time, automatic (Want et al. 2013).

### Statistical analysis

Office 2016 software was used to draw the figures, SPSS Statistics v20.0 (IBM Corp., USA) was used for analysis of variance (ANOVA) or *t*-test on the data, the experiment was repeated three times, the average of the measured results were taken and the data were expressed as the mean ± SD.

## RESULTS

### Antibacterial activity of the *Acer truncatum* leaf extract against *S. aureus in vitro*

According to Table 1, the ethyl acetate extraction phase and saturated *n*-butanol extraction phase of the ethanolic extract from the *Acer truncatum* leaves tested by the K-B method have certain antibacterial effects on *S. aureus*, whereas the petroleum ether extraction exhibits no antibacterial effect. The inhibition zones of the ethyl acetate extraction phase and saturated *n*-butanol extraction phase are 12.77 ± 0.67 mm and 12.37 ± 0.81 mm, respectively. The results indicate that the ethyl acetate extraction phase and saturated *n*-butanol extraction phase of ethanolic extract from the *Acer truncatum* leaves have moderately sensitive antibacterial activities against *S. aureus in vitro*.

**Table 1:** The antibacterial results of various extraction phases of the *Acer truncatum* leaf extract by the disc diffusion method (*n* = 3)

Various extraction phases	Strain
	*S. aureus*
Petroleum ether extraction phase (mean ± SD) mm	–
Ethyl acetate extraction phase (mean ± SD) mm	12.77 ± 0.67
Saturated *n*-butanol extraction phase (mean ± SD) mm	12.37 ± 0.81

### MIC and MBC of the *Acer truncatum* leaf extract against *S. aureus*

**Figure 1:**
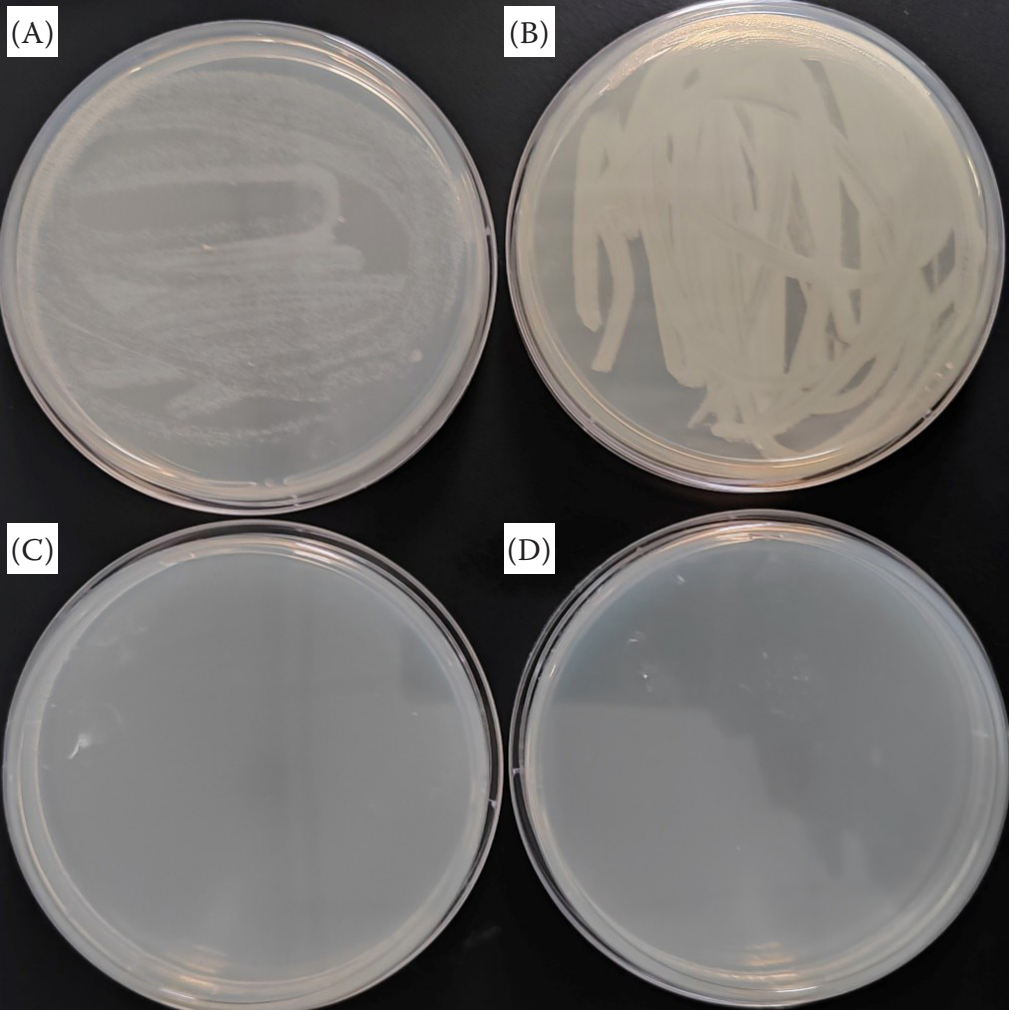
The antibacterial effect of the MBC of *Acer truncatum* leaf extract on *S. aureus* (A) 3.125 mg/ml; (B) Control group; (C) 6.25 mg/ml; (D) Blank control MBC = minimum bactericidal concentration

As shown in Tables 2 and 3, for *S. aureus*, when the concentration of the *n*-butanol extraction fraction of the *Acer truncatum* leaf extract is 3.125 mg/ml, the liquid culture medium was turbid, indicating that there is obvious growth of* S. aureus*. However, when the mass concentration is 6.25 mg/ml, the culture medium is clear, indicating that there is no obvious growth of the strain. Therefore, the MIC of the *n*-butanol extraction fraction against the *S. aureus* is determined as 3.125 mg/ml. According to Table 4 and Figure 1, the MBC of the *n*-butanol extraction fraction against the *S. aureus* is also determined as 6.25 mg/ml.

**Table 2:** The MIC of the *n*-butanol extract of the *Acer truncatum* leaves against *S. aureus*

	Concentration (mg/ml)										MIC (mg/ml)
	Blank	50.000	25.000	12.500	6.250	3.125	1.563	0.781	0.391	0.000	
Growing states	–	–	–	–	–	–	+	+	+	+	3.125

(–) = antibacterial activity; (+) = bacterial growth; MIC = minimum inhibitory concentration

**Table 3:** The inhibitory effect (OD_600 n__m_) of different concentrations of the *n*-butanol extract of *Acer truncatum* leaves on *S. aureus*

OD_600 nm_	Concentration (mg/ml)						
	Blank	6.25	3.125	1.563	0.781	0.391	0.000
Mean ± SD	0.049 ± 0.002^a^	0.051 ± 0.003^a^	0.095 ± 0.007^b^	0.117 ± 0.043^bc^	0.143 ± 0.013^c^	0.193 ± 0.014^d^	0.310 ± 0.002^e^

Comparison between groups

^a–e^Different lowercase letters on the shoulder of the same row indicate significant differences (*P *< 0.05), while the same lowercase letters indicate no significant differences (*P *> 0.05)

OD = optical density

**Table 4:** The MBC of the *n*-butanol extract of the *Acer truncatum* leaves against *S. aureus*

	Concentration (mg/ml)										MBC (mg/ml)
	Blank	50.000	25.000	12.500	6.250	3.125	1.563	0.781	0.391	0.000	
Growing states	–	–	–	–	–	+	+	+	+	+	6.250

(–) = antibacterial activity; (+) = bacterial growth; MBC = minimum bactericidal concentration

### Effect of the *Acer truncatum* leaf extract on the growth of *S. aureus*

**Figure 2:**
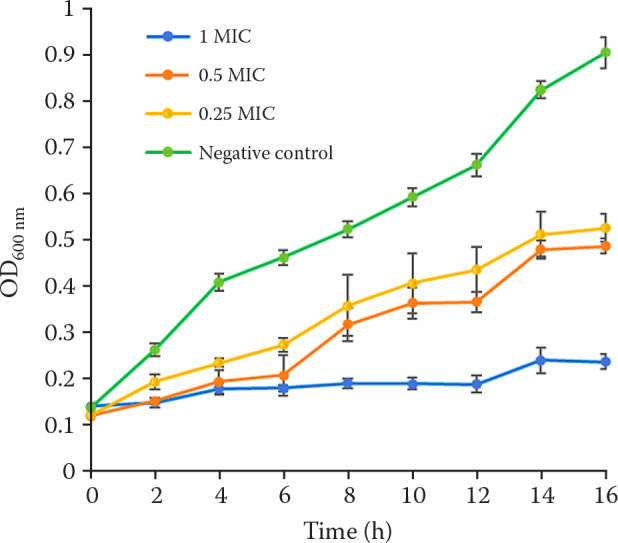
Effect of the *Acer truncatum* leaf extract on the growth of *S. aureus*

As can be seen from Figure 2, the bacterial strain in the control group grew rapidly within 16 h, with an OD_600 nm_ of 0.901 at 16 hours. The growth of the bacterial strain was significantly inhibited by the addition of extracts at different concentrations, and the OD values at the same time were significantly lower than those in the control group. When the concentration of the extract added reached 1 MIC, the growth of the bacterial strain was completely inhibited, and its OD_600 nm_ did not change significantly within 16 hours. This indicates that when the concentration of *Acer truncatum* leaf extract reaches 1 MIC, it can effectively inhibit the growth and reproduction of *S. aureus* in the liquid culture.

### Effect of the *Acer truncatum* leaf extract on the cell membrane integrity of *S. aureus*

**Figure 3:**
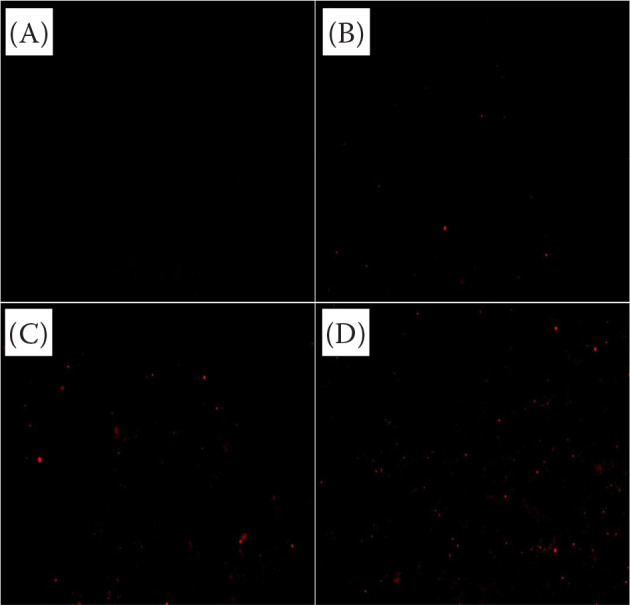
Effects of different concentrations of the *Acer truncatum* leaf extract on the cell membrane integrity of *S. aureus* (fluorescence microscope) (A) Negative control; (B) 0.5 MIC; (C) 1 MIC; (D) 2 MIC MIC = minimum inhibitory concentration

As a membrane-impermeable fluorescent dye, PI has a unique mechanism of action (Perrine-Walker and Le 2021). When the integrity of the cell membrane is damaged, it can penetrate the cell membrane, bind to genetic material, and emit red fluorescence. Therefore, the integrity of the cell membrane can be reflected by detecting PI fluorescence through a fluorescence microscope. As shown in Figure 3, there was no observable fluorescence in the control group after PI staining. Sporadic red fluorescence was emitted from the *S. aureus* group treated with 0.5 MIC metabolites for 4 h, and the red fluorescence gradually increased with the increase in the extract concentration. When the mass concentration reached 2 MIC, a large amount of red fluorescence was observed, indicating that after being treated with the extract, the cell membrane of *S. aureus* was destroyed, allowing a large amount of PI to enter the cell and bind to genetic material, emitting red fluorescence.

### Compositional analysis of the *Acer truncatum* leaf extract

**Figure 4:**
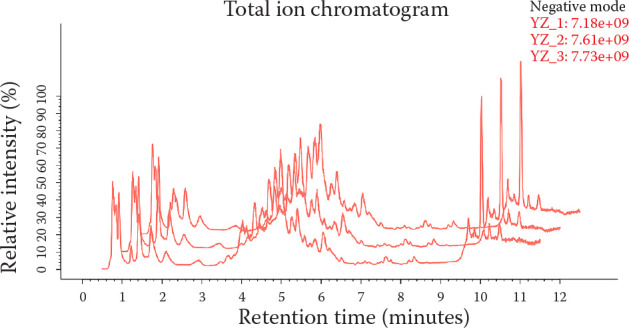
Total ion chromatogram (TIC) in negative ion mode (*n* = 3)

**Figure 5:**
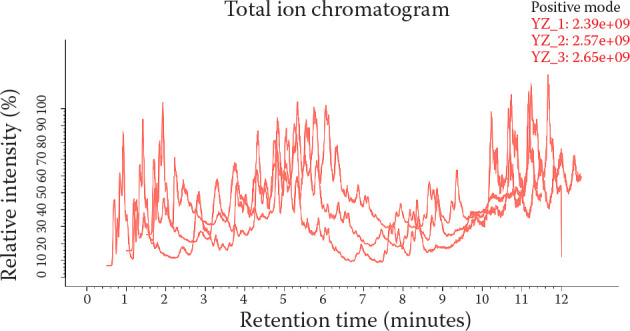
Total ion chromatogram (TIC) in positive ion mode (*n* = 3)

LC-MS/MS identified 179 low-molecular-weight metabolites in the extract (Figures 4 and 5). These peaks mainly include low-molecular-weight phenolic compounds, alkaloids, flavonoids, organic acids, amino acids, sugars, lipids, and sugar alcohols [Electronic Supplementary Material (ESM) Table S1]. Among them, betaine (27.189%), 3,4-dihydroxymandelic acid (16.112%), quercitrin (14.768%), chlorogenic acid (8.778%), and neochlorogenic acid (4.452%) had the highest content. There were 10 compounds with a relative content greater than 1% (Table 5).

**Table 5:** Main components of the *Acer truncatum* leaf extract

ID	Compound	Mz	*Rt* (s)	Formula	Relative content (%)
M117T644	Betaine	116.928	644.4	C5H11NO2	27.189
M183T240_2	3,4-Dihydroxymandelic acid	183.029 7	240.3	C8H8O5	16.112
M447T300_2	Quercitrin	447.092	299.9	C21H20O11	14.768
M353T84_2	Chlorogenic acid	353.087 8	84	C16H18O9	8.778
M353T55_2	Neochlorogenic acid	353.087 8	55.2	C16H18O9	4.452
M319T285_1	Myricetin	319.045 3	284.7	C15H10O8	3.495
M197T355	Vanillylmandelic acid	197.046	355	C9H10O5	2.037
M150T681	d-Ribose	149.994 6	681	C5H10O5	1.670
M595T251_3	Isovitexin 2''-O-beta-d-glucoside	595.163 7	251	C27H30O15	1.623
M104T49	Choline	104.107 2	48.5	C5H14NO	1.566
M303T351	Quercetin	303.050 6	351.3	C15H10O7	0.991
M277T582	Alpha-Linolenic acid	277.217 8	582.3	C18H30O2	0.804
M124T634	Picolinic acid	124.087 5	633.7	C6H5NO2	0.799
M256T685	Palmitic acid	256.266 5	685.2	C16H32O2	0.755
M124T602	Nicotinic acid	124.086 2	602.4	C6H5NO2	0.736
M376T459_2	Riboflavin	376.259	459.1	C17H20N4O6	0.724
M282T631	Oleic acid	282.279 6	630.6	C18H34O2	0.611
M145T633	Anabasine	144.981 1	633.5	C10H14N2	0.573
M173T46	Shikimic acid	173.047	45.8	C7H10O5	0.509
M123T73	Niacinamide	123.055 2	72.8	C6H6N2O	0.482
M289T237	Catechin	289.071 9	237.3	C15H14O6	0.446
M137T101	2-Methylbenzoic acid	137.06	101.1	C8H8O2	0.432
M284T623	Octadecanamide	284.286 5	623.4	C18H37NO	0.339
M116T53_2	l-Proline	116.070 7	52.5	C5H9NO2	0.291
M279T614	Bovinic acid	279.233 2	614.1	C18H32O2	0.276
M287T378	Fisetin	287.056 3	378.3	C15H10O6	0.263
M153T237	2-Pyrocatechuic acid	153.02	236.9	C7H6O4	0.240
M627T231_3	Delphinidin 3,7-di-O-beta-d-glucoside	627.159 9	231.1	C27H31O17	0.232
M166T83_2	Benzocaine	166.086 5	82.9	C9H11NO2	0.222
M243T174	3-Methoxy-4',5-dihydroxy-trans-stilbene	243.087 6	173.6	C15H14O3	0.212
					
Total					91.627

## DISCUSSION

*S. aureus* is a crucial pathogenic species in veterinary medicine. It poses a significant threat due to its harmful impact on animal health and the potential for zoonotic transmission. This not only endangers animal well-being, but also leads to substantial economic losses in livestock production. Consequently, growing attention is being directed towards both livestock and companion animals regarding this pathogen (Peton and Le Loir 2014; Costa et al. 2022). Natural medicinal plants combat bacterial growth through multifaceted mechanisms. These include damaging the bacterial cell walls/membranes, disrupting metabolism/reproduction cycles, and modulating host immune/anti-inflammatory responses. Additionally, they target biofilm regulators, inhibit secretion systems, suppress the virulence gene expression, reduce the pathogenicity, and block the respiratory chain activity. Collectively, these actions minimise the development of drug resistance (Garg and Roy 2020; Bittner Fialova et al. 2021). This study indicates that the extract of *Acer truncatum* leaves has a significant inhibitory effect on *S. aureus*. The MIC of the extract against *S. aureus* was 3.125 mg/ml. At 0.25 MIC, growth inhibition was evident, while 1 MIC completely suppressed growth (OD_600 nm_ remained stable over 24 hours). The fluorescence microscopy of PI-stained *S. aureus* revealed concentration-dependent membrane damage. At 2 MIC, severe membrane damage and widespread bacterial death occurred. PI fluorescent dye selectively enters the bacteria with damaged cell walls/membranes and bind to nucleic acids, emitting red fluorescence (Stiefel et al. 2015; Rosenberg et al. 2019). Therefore, it is speculated that the antibacterial mechanism of the extract against *S. aureus* may be due to the increase in the permeability of the cell membrane of *S. aureus* with the increase in the extract concentration, which destroys the integrity of the cell membrane, causes the influx of a large number of exogenous substances and the leakage of cytoplasm, and leads to intracellular osmotic pressure disorder, making the bacteria unable to grow and reproduce normally. Some studies have shown that antibiotics exert antibacterial effects by destroying the cell membranes of pathogenic bacteria (Berti et al. 2015). The cell membrane is a supermolecular structure and an important channel for material and energy exchange as well as information transmission between bacteria and the environment (Song et al. 2021). In this experiment, the extract of *Acer truncatum* leaves can destroy the integrity of the *S. aureus* cell membrane, which is consistent with previous research results on the antibacterial mechanism of traditional Chinese medicinal materials such as *Forsythia suspense*, *Astragalus membranaceus* and *Citrus medica* var. *sarcodactylis* (Wang et al. 2021).

Due to its rapid, high-resolution, and high-sensitivity features, the LC-MS technology has been widely used in the analysis of natural small molecules. These compounds typically possess structural characteristics that generate diagnostic fragments during MS/MS dissociation (Fu et al. 2019; Yang et al. 2022b). In this study, LC-MS/MS analyses of *Acer truncatum* leaf extract identified 179 compounds in total. The five most abundant components were betaine, 3,4-dihydroxymandelic acid, quercitrin, chlorogenic acid, and neochlorogenic acid, collectively accounting for 71.30% of the total content. Among these, 3,4-dihydroxymandelic acid and chlorogenic acid are polyphenolic compounds. Antibacterial activity has been linked to phenolic compound content in previous studies (Nunes et al. 2017). The reported antibacterial substances include quercitrin, chlorogenic acid, neochlorogenic acid, myricetin, and others. Fan et al. (2022) reviewed global *Acer truncatum* studies (1949–2021), documenting 288 compounds such as polyphenols, organic acids, lipids, and biological volatile organic compounds.

Betaine has the highest relative content in the extract, accounting for 27.189%. Betaine, also known as trimethylglycine, is widely distributed in animals, plants, and microorganisms, and exhibits anti-inflammatory effects against numerous diseases (Zhao et al. 2018). However, no antibacterial activity has been reported for betaine. Quercitrin is a natural flavonoid glycoside compound commonly used as a dietary ingredient and supplement. To date, its extensive biological activities have been revealed, including antioxidant, anti-inflammatory, antimicrobial, immunomodulatory, analgesic, wound-healing, and vasodilatory effects (Chen et al. 2022). Bismelah et al. (2022) demonstrated that *Scutellaria* extract possesses strong antibacterial activity, with quercitrin as its primary chemical component. Rodriguez-Perez et al. (2016) evaluated the antibacterial activity of *Vaccinium macrocarpon* extract against urinary pathogenic *E. coli*, revealing that its main components – myricetin and quercetin – inhibited *E. coli* virulence by suppressing biofilm formation and reducing bacterial surface hydrophobicity. In our research results, the *Acer truncatum* extract contains myricetin and quercetin, with relative contents of 3.495% and 0.991%, respectively. It has been confirmed that the extract can affect the integrity of the cell membrane of *S. aureus*. Quercetin differs from its aglycone quercetin through the addition of rhamnose via a glycosidic bond at the 3-hydroxy position group of quercetin (Heim et al. 2002). Liu et al. (2015) found that quercitrin did not affect the growth of *S. aureus*, but it could inhibit sortase A, an enzyme essential for gram-positive bacteria. Sortase A acts as a molecular “glue” that covalently attaches surface proteins to the bacterial cell wall, facilitating crucial functions such as adhesion. By curbing sortase A, quercitrin could disrupts the adhesion of *S. aureus*, thereby reducing the virulence.

Chlorogenic acid is a widely distributed natural compound with many important pharmacological effects that can be found in a variety of plants. As a common natural plant extract, its primary pharmacological activities include antibacterial properties (Miao and Xiang 2020). Xu et al. (2021) demonstrated that *Taraxacum officinale* extract inhibits *S. aureus* growth with a MIC of 12.5 mg/ml, acting by disrupting cell wall and cell membrane integrity. Chlorogenic acid is the extract’s most abundant compound (maximum content: 1.34 mg/g). Yang et al. (2022a) synthesised chitosan-grafted chlorogenic acid (CS-g-CA), which exhibited a MIC of 0.625 mg/ml against *S. aureus*, demonstrating potent antibacterial and anti-biofilm activity.

Additionally, chlorogenic acid has been reported to alleviate *S. aureus*-induced inflammatory responses (Ji et al. 2022). Neochlorogenic acid, a natural polyphenolic compound found in dried fruits and other plants, exhibits excellent antibacterial effects (Kim et al. 2015; Gao et al. 2020; Li et al. 2021). Silva et al. (2022) evaluated the combination of malvidin-3-glucoside and neochlorogenic acid against *S. aureus*. While the combination did not significantly inhibit bacterial growth after 24 h, it effectively reduced biofilm formation and bacterial adhesion, suggesting potential as an anti-adhesion and anti-biofilm agent.

In summary, the *Acer truncatum* extract exhibits significant antibacterial activity against *S. aureus in vitro*, achieving long-term antibacterial effects by disrupting cell membrane integrity. It is postulated that the major components, such as quercitrin, chlorogenic acid, neochlorogenic acid, and myricetin, contribute to this activity. However, the current studies have not delved into the deep control mechanism of effective antibacterial components in the extract on *S. aureus*. Future studies will employ omics approaches to investigate the antibacterial mechanism at the molecular level.

## Supplementary Files

Electronic Supplementary Material (ESM) Table S1

## References

[R1] Berti AD, Baines SL, Howden BP, Sakoulas G, Nizet V, Proctor RA, Rose WE. Heterogeneity of genetic pathways toward daptomycin nonsusceptibility in Staphylococcus aureus determined by adjunctive antibiotics. Antimicrob Agents Chemother. 2015 May;59(5):2799-806. 25733508 10.1128/AAC.04990-14 PMC4394801

[R2] Bismelah NA, Ahmad R, Mohamed Kassim ZH, Ismail NH, Rasol NE. The antibacterial effect of Plectranthus scutellarioides (L.) R.Br. leaves extract against bacteria associated with peri-implantitis. J Tradit Complement Med. 2022 Aug 3;12(6):556-66. 36325238 10.1016/j.jtcme.2022.07.002 PMC9618393

[R3] Bittner Fialova S, Rendekova K, Mucaji P, Nagy M, Slobodnikova L. Antibacterial activity of medicinal plants and their constituents in the context of skin and wound infections, considering european legislation and folk medicine – A review. Int J Mol Sci. 2021 Oct 4;22(19): 10746. 34639087 10.3390/ijms221910746 PMC8509446

[R4] Chen J, Li G, Sun C, Peng F, Yu L, Chen Y, Tan Y, Cao X, Tang Y, Xie X, Peng C. Chemistry, pharmacokinetics, pharmacological activities, and toxicity of Quercitrin. Phytother Res. 2022 Apr;36(4):1545-75. 35253930 10.1002/ptr.7397

[R5] Costa SS, Ribeiro R, Serrano M, Oliveira K, Ferreira C, Leal M, Pomba C, Couto I. Staphylococcus aureus causing skin and soft tissue infections in companion animals: Antimicrobial resistance profiles and clonal lineages. Antibiotics (Basel). 2022 Apr 29;11(5):599. 35625243 10.3390/antibiotics11050599 PMC9137735

[R6] Deepak SJ, Kannan P, Savariraj WR, Ayyasamy E, Tuticorin Maragatham Alagesan SK, Ravindran NB, Sundaram S, Mohanadasse NQ, Kang Q, Cull CA, Amachawadi RG. Characterization of Staphylococcus aureus isolated from milk samples for their virulence, biofilm, and antimicrobial resistance. Sci Rep. 2024 Oct 27;14(1):25635. 39465266 10.1038/s41598-024-75076-y PMC11514165

[R7] Deng M, Wang Y, Chen G, Liu J, Wang Z, Xu H. Poly-l-lysine-functionalized magnetic beads combined with polymerase chain reaction for the detection of Staphylococcus aureus and Escherichia coli O157:H7 in milk. J Dairy Sci. 2021 Dec;104(12):12342-52. 34482981 10.3168/jds.2021-20612

[R8] Ekiert HM, Szopa A. Biological activities of natural products. Molecules. 2020 Dec 7;25:5769. 33297511 10.3390/molecules25235769 PMC7730830

[R9] Fan H, Sun L, Yang L, Zhou J, Yin P, Li K, Xue Q, Li X, Liu Y. Assessment of the bioactive phenolic composition of Acer truncatum seed coat as a byproduct of seed oil. Ind Crops Prod. 2018 Aug;118:11-9.

[R10] Fan Y, Lin F, Zhang R, Wang M, Gu R, Long C. Acer truncatum Bunge: A comprehensive review on ethnobotany, phytochemistry and pharmacology. J Ethnopharmacol. 2022 Jan 10;282:114572. 34487848 10.1016/j.jep.2021.114572

[R11] Fu T, Houel E, Amusant N, Touboul D, Genta-Jouve G, Della-Negra S, Fisher GL, Brunelle A, Duplais C. Biosynthetic investigation of γ-lactones in Sextonia rubra wood using in situ TOF-SIMS MS/MS imaging to localize and characterize biosynthetic intermediates. Sci Rep. 2019 Feb 13;9(1):1928. 30760744 10.1038/s41598-018-37577-5 PMC6374367

[R12] Gao F, Wang T, Xiao J, Huang G. Antibacterial activity study of 1,2,4-triazole derivatives. Eur J Med Chem. 2019 Jul 1; 173:274-81. 31009913 10.1016/j.ejmech.2019.04.043

[R13] Gao XH, Zhang SD, Wang LT, Yu L, Zhao XL, Ni HY, Wang YQ, Wang JD, Shan CH, Fu YJ. Anti-inflammatory effects of neochlorogenic acid extract from mulberry leaf (Morus alba L.) against LPS-stimulated inflammatory response through mediating the AMPK/Nrf2 signaling pathway in A549 cells. Molecules. 2020 Mar 18;25(6):1385. 32197466 10.3390/molecules25061385 PMC7144357

[R14] Garg S, Roy A. A current perspective of plants as an antibacterial agent: A review. Curr Pharm Biotechnol. 2020; 21(15):1588-602. 32568018 10.2174/1389201021666200622121249

[R15] Giao MS, Wilks SA, Azevedo NF, Vieira MJ, Keevil CW. Validation of SYTO 9/propidium iodide uptake for rapid detection of viable but noncultivable Legionella pneumophila. Microb Ecol. 2009 Jul;58(1):56-62. 19043657 10.1007/s00248-008-9472-x

[R16] He X, Li DZ, Tian B. Diversity in seed oil content and fatty acid composition in Acer species with potential as sources of nervonic acid. Plant Divers. 2020 Oct 31;43(1):86-92. 33778229 10.1016/j.pld.2020.10.003 PMC7987630

[R17] Heim KE, Tagliaferro AR, Bobilya DJ. Flavonoid antioxidants: Chemistry, metabolism and structure-activity relationships. J Nutr Biochem. 2002 Oct;13(10):572-84. 12550068 10.1016/s0955-2863(02)00208-5

[R18] Ji Q, Zhang M, Wang Y, Chen Y, Wang L, Lu X, Bai L, Wang M, Bao L, Hao H, Wang Z. Protective effects of chlorogenic acid on inflammatory responses induced by Staphylococcus aureus and milk protein synthesis in bovine mammary epithelial cells. Microb Pathog. 2022 Oct;171:105726. 35995255 10.1016/j.micpath.2022.105726

[R19] Kim M, Choi SY, Lee P, Hur J. Neochlorogenic acid inhibits lipopolysaccharide-induced activation and pro-inflammatory responses in BV2 microglial cells. Neurochem Res. 2015 Sep;40(9):1792-8. 26152332 10.1007/s11064-015-1659-1

[R20] Li Y, Kong F, Wu S, Song W, Shao Y, Kang M, Chen T, Peng L, Shu Q. Integrated analysis of metabolome, transcriptome, and bioclimatic factors of Acer truncatum seeds reveals key candidate genes related to unsaturated fatty acid biosynthesis, and potentially optimal production area. BMC Plant Biol. 2024 Apr 16;24(1):284. 38627650 10.1186/s12870-024-04936-6 PMC11020666

[R21] Li Y, Yu X, Deng L, Zhou S, Wang Y, Zheng X, Chu Q. Neochlorogenic acid anchors MCU-based calcium overload for cancer therapy. Food Funct. 2021 Sep 17;12(22): 11387-98. 34672304 10.1039/d1fo01393a

[R22] Liao F, He J, Li R, Hu Y. Endophytic fungus UJ3-2 from Urtica fissa: Antibacterial activity and mechanism of action against Staphylococcus aureus. Molecules. 2024 Oct 13;29(20):4850. 39459217 10.3390/molecules29204850 PMC11510654

[R23] Liu B, Chen F, Bi C, Wang L, Zhong X, Cai H, Deng X, Niu X, Wang D. Quercitrin, an inhibitor of Sortase A, interferes with the adhesion of Staphylococcal aureus. Molecules. 2015 Apr 13;20(4):6533-43. 25871372 10.3390/molecules20046533 PMC6272417

[R24] Miao M, Xiang L. Pharmacological action and potential targets of chlorogenic acid. Adv Pharmacol. 2020 Jan;87: 71-88. 32089239 10.1016/bs.apha.2019.12.002

[R25] Nunes R, Pasko P, Tyszka-Czochara M, Szewczyk A, Szlosarczyk M, Carvalho IS. Antibacterial, antioxidant and anti-proliferative properties and zinc content of five south Portugal herbs. Pharm Biol. 2017 Dec;55(1):114-23. 27925492 10.1080/13880209.2016.1230636 PMC7011791

[R26] Perrine-Walker F, Le K. Propidium iodide enabled live imaging of Pasteuria sp.-Pratylenchus zeae infection studies under fluorescence microscopy. Protoplasma. 2021 Mar; 258(2):279-87. 33070241 10.1007/s00709-020-01567-0

[R27] Peton V, Le Loir Y. Staphylococcus aureus in veterinary medicine. Infect Genet Evol. 2014 Jan;21:602-15. 23974078 10.1016/j.meegid.2013.08.011

[R28] Rodriguez-Perez C, Quirantes-Pine R, Uberos J, Jimenez-Sanchez C, Pena A, Segura-Carretero A. Antibacterial activity of isolated phenolic compounds from cranberry (Vaccinium macrocarpon) against Escherichia coli. Food Funct. 2016 Mar;7(3):1564-73. 26902395 10.1039/c5fo01441g

[R29] Rosenberg M, Azevedo NF, Ivask A. Propidium iodide staining underestimates viability of adherent bacterial cells. Sci Rep. 2019 Apr 24;9(1):6483. 31019274 10.1038/s41598-019-42906-3 PMC6482146

[R30] Lan SB. Research overview and development prospect of acer linn plants. Fore by-Prod Spec Chi. 2019;162(5):84-9.

[R31] Silva S, Costa EM, Machado M, Morais R, Calhau C, Pintado M. Antiadhesive and antibiofilm effect of malvidin-3-glucoside and malvidin-3-glucoside/neochlorogenic acid mixtures upon staphylococcus. Metabolites. 2022 Nov 3; 12(11):1062. 36355145 10.3390/metabo12111062 PMC9694786

[R32] Song J, GuangXin L, HanShen W, Meng L. [Antimicrobial activity and mechanism of sanguinarine against Streptococcus aga-lactiae]. Chinese J Vet Sci. 2021;41(6):1067-73. Chinese.

[R33] Stiefel P, Schmidt-Emrich S, Maniura-Weber K, Ren Q. Critical aspects of using bacterial cell viability assays with the fluorophores SYTO9 and propidium iodide. BMC Microbiol. 2015 Feb 18;15:36. 25881030 10.1186/s12866-015-0376-x PMC4337318

[R34] Tan S, Cho K, Nodwell JR. A defect in cell wall recycling confers antibiotic resistance and sensitivity in Staphylococcus aureus. J Biol Chem. 2022 Oct;298(10):102473. 36089064 10.1016/j.jbc.2022.102473 PMC9547203

[R35] Tasneem U, Mehmood K, Majid M, Ullah SR, Andleeb S. Methicillin resistant Staphylococcus aureus: A brief review of virulence and resistance. J Pak Med Assoc. 2022 Mar;72(3):509-15. 35320234 10.47391/JPMA.0504

[R36] Troscianczyk A, Nowakiewicz A, Kasela M, Malm A, Tracz AM, Hahaj-Siembida A, Osinska M, Gula S, Jankowiak I. Multi-host pathogen Staphylococcus aureus – Epidemiology, drug resistance and occurrence in humans and animals in Poland. Antibiotics (Basel). 2023 Jun 30;12(7):1137. 37508233 10.3390/antibiotics12071137 PMC10376275

[R37] Wang Q, Zhang F, Wang Z, Chen H, Wang X, Zhang Y, Li S, Wang H. Evaluation of the Etest and disk diffusion method for detection of the activity of ceftazidime-avibactam against Enterobacterales and Pseudomonas aeruginosa in China. BMC Microbiol. 2020 Jun 29;20(1):187. 32600252 10.1186/s12866-020-01870-z PMC7325266

[R38] Wang XH, Guo R, Nie XB, Chen HM, Zhi WW, Tian ML, Liu F. [Antibacterial activity of Citri Sarcodactylis Fructus and its antibacterial mechanism against Staphylococcus aureus]. Chin J Antibiot. 2021;46:437-41. Chinese.

[R39] Want EJ, Masson P, Michopoulos F, Wilson ID, Theodoridis G, Plumb RS, Shockcor J, Loftus N, Holmes E, Nicholson JK. Global metabolic profiling of animal and human tissues via UPLC-MS. Nat Protoc. 2013 Jan;8(1):17-32. 23222455 10.1038/nprot.2012.135

[R40] Xu P, Xu XB, Khan A, Fotina T, Wang SH. Antibiofilm activity against Staphylococcus aureus and content analysis of Taraxacum Officinale phenolic extract. Pol J Vet Sci. 2021 Jun;24(2):243-51. 34250777 10.24425/pjvs.2021.137659

[R41] Yang X, Lan W, Xie J. Antimicrobial and anti-biofilm activities of chlorogenic acid grafted chitosan against Staphylococcus aureus. Microb Pathog. 2022a Dec;173(Pt A): 105748. 36064104 10.1016/j.micpath.2022.105748

[R42] Yang YL, Adel Al-Mahdy D, Wu ML, Zheng XT, Piao XH, Chen AL, Wang SM, Yang Q, Ge YW. LC-MS-based identification and antioxidant evaluation of small molecules from the cinnamon oil extraction waste. Food Chem. 2022b Jan 1;366:130576. 34348222 10.1016/j.foodchem.2021.130576

[R43] Zaatout N, Ayachi A, Kecha M. Staphylococcus aureus persistence properties associated with bovine mastitis and alternative therapeutic modalities. J Appl Microbiol. 2020 Nov;129(5):1102-19. 32416020 10.1111/jam.14706

[R44] Zelena E, Dunn WB, Broadhurst D, Francis-McIntyre S, Carroll KM, Begley P, O’Hagan S, Knowles JD, Halsall A; HUSERMET Consortium; Wilson ID, Kell DB. Development of a robust and repeatable UPLC-MS method for the long-term metabolomic study of human serum. Anal Chem. 2009 Feb 15;81(4):1357-64. 19170513 10.1021/ac8019366

[R45] Zhao G, He F, Wu C, Li P, Li N, Deng J, Zhu G, Ren W, Peng Y. Betaine in inflammation: Mechanistic aspects and applications. Front Immunol. 2018 May 24;9:1070. 29881379 10.3389/fimmu.2018.01070 PMC5976740

[R46] Zhou H, Du W, Ouyang D, Li Y, Gong Y, Yao Z, Zhong M, Zhong X, Ye X. Simple and accurate genomic classification model for distinguishing between human and pig Staphylococcus aureus. Commun Biol. 2024 Sep 18; 7(1):1171. 39294434 10.1038/s42003-024-06883-2 PMC11410946

